# Polystyrene microplastics increase microbial release of marine Chromophoric Dissolved Organic Matter in microcosm experiments

**DOI:** 10.1038/s41598-018-32805-4

**Published:** 2018-10-02

**Authors:** Luisa Galgani, Anja Engel, Claudio Rossi, Alessandro Donati, Steven A. Loiselle

**Affiliations:** 1 Department of Biotechnology, Chemistry and Pharmacy, University of Siena, Siena, Italy; 2 GEOMAR – Helmholtz Centre for Ocean Research Kiel, Kiel, Germany

## Abstract

About 5 trillion plastic particles are present in our oceans, from the macro to the micro size. Like any other aquatic particulate, plastics and microplastics can create a micro-environment, within which microbial and chemical conditions differ significantly from the surrounding water. Despite the high and increasing abundance of microplastics in the ocean, their influence on the transformation and composition of marine organic matter is largely unknown. Chromophoric dissolved organic matter (CDOM) is the photo-reactive fraction of the marine dissolved organic matter (DOM) pool. Changes in CDOM quality and quantity have impacts on marine microbial dynamics and the underwater light environment. One major source of CDOM is produced by marine bacteria through their alteration of pre-existing DOM substrates. In a series of microcosm experiments in controlled marine conditions, we explored the impact of microplastics on the quality and quantity of microbial CDOM. In the presence of microplastics we observed an increased production of CDOM with changes in its molecular weight, which resulted from either an increased microbial CDOM production or an enhanced transformation of DOM from lower to higher molecular weight CDOM. Our results point to the possibility that marine microplastics act as localized hot spots for microbial activity, with the potential to influence marine carbon dynamics.

## Introduction

Microplastics are operationally defined as plastic particles with dimensions ranging from a few nanometers up to 5 millimeters^1^. They are released directly into the environment (primary microplastics) as cosmetic microbeads, pellets, granules, or derived from the weathering of larger plastic items (secondary microplastics). The global annual release of plastic into the ocean is estimated to be 8 million metric tons per year^2^. In its journey to and within the sea, larger plastic items undergo multiple breakdown processes and major efforts have been made to better understand the quantity and quality of microplastics in the marine environment^3^. Furthermore, the comparison of the land-to-sea fluxes to *in-situ* concentrations suggests the presence of in-ocean removal mechanisms^4^, with 5 trillion plastic particles present in today’s ocean which however underestimates the abundance of microplastics compared to fragmentation models^5^.

The most abundant microplastics polymers found in marine surface waters are low-density polyolefines (polyethylene, polypropylene) and polystyrene^6,7^. Plastics and microplastics in general can act as a physical host for a wide variety of surface-attached phytoplankton, bacteria and invertebrates^8–11^. Microbial attachment on organic and inorganic particles is a common phenomenon in aquatic ecosystems^12^. As a function of the available substrate and organisms present, the first step in the microbial attachment on plastic debris starts with the formation of a conditioning film able to modify material-specific surface properties and influence the colonizing community^13^. Once established, the first coating may facilitate the adhesion of other microorganisms towards the formation of a more complex biofilm^9^, a matrix of self-produced extracellular organic matter that provides mechanical stability, mediates microbial adhesion to surfaces and allows for microbial metabolism by embedding extracellular enzymes^14^.

With their increased concentrations in the aquatic environment, microplastics may become a vector for carbon transport by favoring the marine biological pump^15^.

In general, photosynthetic fixation of inorganic carbon into organic matter in surface waters and its export to the deep ocean is a primary carbon pump in marine ecosystems. However, about half of oceanic primary production is channeled into the dissolved phase as Dissolved Organic Matter (DOM), which is present in a continuum of sizes and reactivities, including colloidal phases. Bacterial interaction with this pool creates activity hotspots that can host high microbial diversity and distinct biogeochemical processes^16^.

Chromophoric Dissolved Organic Matter (CDOM), the principal light-absorbing fraction of the DOM pool with respect to UV (100–400 nm) and visible radiation (400–700 nm), is an important tracer of water masses, a driver of microbial carbon dynamics and regulator of the underwater light environment with repercussions on primary production^17^. CDOM comprises 20–70% of DOM in the ocean, even though it is highly photo-reactive^18^. CDOM photolysis facilitates the availability of low molecular weight carbon compounds for microbial growth^19^. However, photo-lability will depend on CDOM source and prior exposure. Bacterial CDOM may be more refractory than CDOM from allochthonous sources and its photolysis may generate material resistant to any further biological utilization^20,21^.

The increased presence of microplastics as resistant organic particles in the marine ecosystem may have important impacts simply by acting as a substrate for microbial growth. While recent findings have shown that carbon leaching from plastics may stimulate microbial activity^22^, also by acting as a substrate for organic matter release microplastics would be interfering with organic matter cycling in the oceans.

In two sets of controlled laboratory experiments, referred to as experimental set up 1 and 2 (ES1 and ES2), we explored the impacts of combined photo-and microbial alterations on complex DOM substrates in the presence of inert polystyrene microplastics. This study indicates that the presence of microplastic particles may increase the microbial release of CDOM. If extrapolated to the field, these results suggest that the increasing abundance of microplastics in our oceans may modify the marine cycling of organic matter.

## Results

In two different sets of experiments, ES1 and ES2, we used the organic material (DOM) from filtered marine phytoplankton exudates, removing phytoplankton cells by filtration but keeping bacteria in the systems. The effects of microplastics on the microbial or photochemical cycling of phytoplankton-derived DOM was measured at regular intervals over several days, using 10-cm path length cylindrical quartz cuvettes (microcosms). Half of the microcosms were treated with high concentrations of transparent 30 µm diameter polystyrene spheres, thereafter named as MP, and compared to controls (C) without microplastic addition. Part of the samples (both C and MP) were kept in the dark while other samples were exposed to pseudo-sunlight conditions. An initial blank control experiment in Milli-Q water was performed to exclude the possibility of absorbance or leachates from the polystyrene spheres.

ES1 consisted of three replicate experiments (ES1a, ES1b and ES1c) using exudates of three different marine algae. Six cuvettes were amended with microplastics at a concentration of 2200 particles per liter, comparable to concentrations in marine sediments and sea ice^23,24^. In ES2, we focused on potential impacts of microplastics on DOM cycling with respect to photochemical processes with two experiments in series, first in the light and subsequently in the dark. For ES2, higher concentrations of microplastics (92000 particlesL^−1^) were used. In both ES1 and ES2 changes in CDOM, dissolved organic carbon (DOC) and bacterial abundance were measured at fixed intervals over the experimental periods. Measuring UV-Visible spectra directly from the microcosms allowed us to compare changes in real time between MP treatments and controls.

We used standard polystyrene spheres that did not contain additives and did not leach any optically active components within the time frame of our experiments. The influence of the particles on CDOM absorbance was removed by correcting all spectra for scattering at 700 nm, and checked in the blank-control experiment in Milli-Q water. All microcosm data were normalized to initial conditions to allow for comparison between experiments and microcosms.

**Figure 1:**
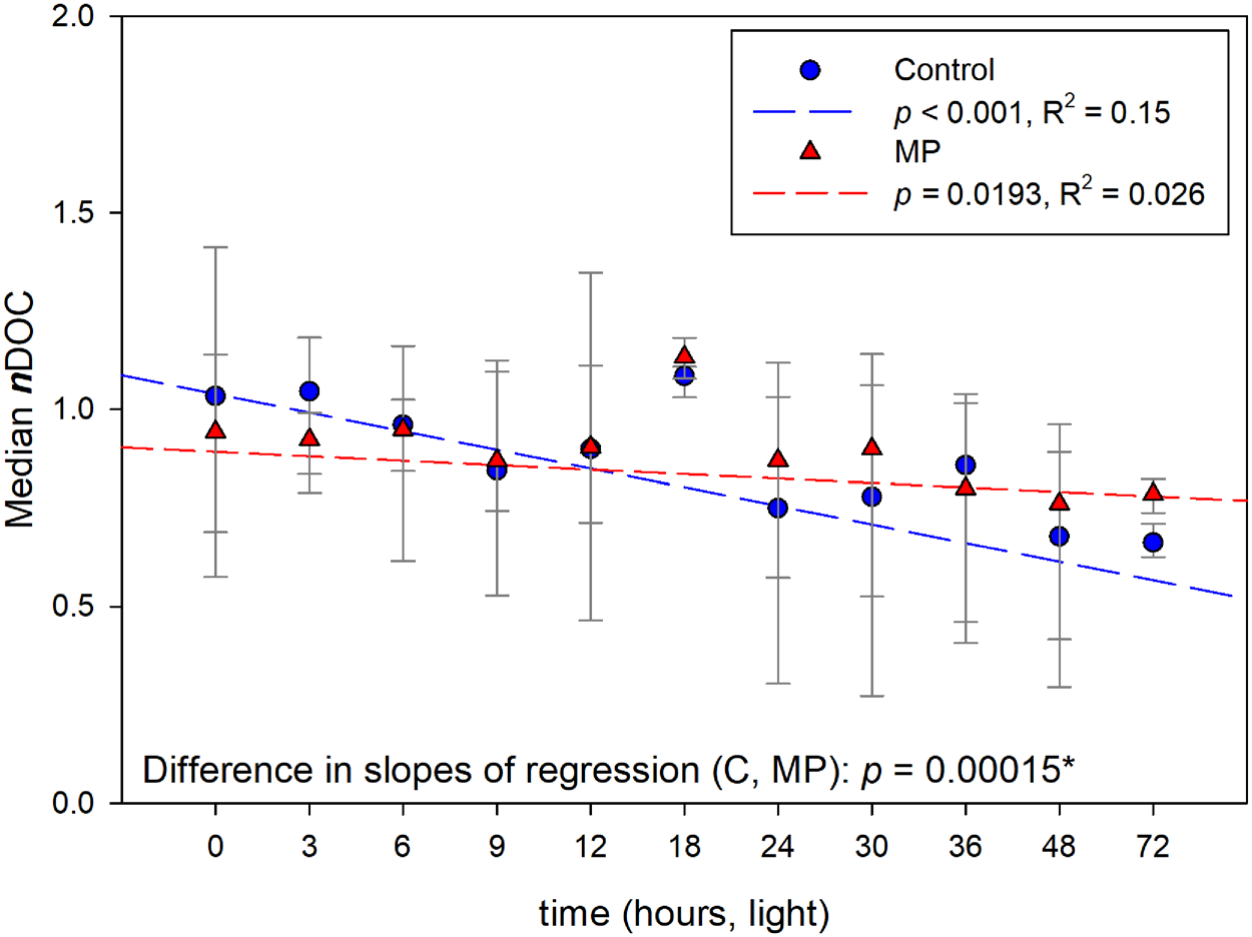
Changes in estimated DOC with increasing exposure to light in ES1. Data wee normalized with each dot representing the median of all samples at that exposure time.

In ES1a,b,c, the three monoculture experiments with DOM characteristics similar to oligotrophic marine waters showed a decrease of DOC concentration with increased light exposure time (Table S1). The DOC loss rate over time was slightly higher in the control (C) with respect to the treated samples (MP) (Fig. 1, Table S1). The loss of DOC upon light exposure was expected and observed in both C and MP samples, regardless of the starting organic material (ES1a,b and c). By using normalized data from the three experiments ES1a, ES1b and ES1c and by considering a linear regression with increased exposure time, the differences in the slopes of the regression lines between C and MP (Fig. 1, *p* = 0.00015) indicate a less pronounced DOC loss in MP samples. In the second set of experiments (ES2) with a heterogeneous phytoplankton culture and higher concentrations of exudates and microplastics, DOC decreased again with exposure time in the light, but with no clear differences between C and MP (Table S1, Fig. S1). In the dark, DOC increased in both C and MP (Fig. S1).

In ES1 bacteria cell numbers did not show any discernable linear trend over time (*p* > 0.05) and no significant differences were observed between C and MP (Fig. S2, on normalized data). Cell number averages were 2.6 ± 0.6 × 10^6^ cells mL^−1^ (ES 1a), 1.9 ± 1.2 × 10^6^ cells mL^−1^ (ES 1b), and 2.1 ± 0.2 × 10^6^ cells mL^−1^ (ES 1c). In ES2, net changes in dissolved oxygen concentrations were used as a proxy for net microbial activity (bacteria and phytoplankton). Increases in dissolved oxygen concentrations in light conditions occurred in both treatments (C and MP) indicating a net autotrophic production. Initial concentrations of 6.34 ± 0.05 mg O_2_ L^−1^ (C) and 6.61 ± 0.04 mg O_2_ L^−1^ (MP) increased to 12.34 ± 0.08 mg O_2_ L^−1^ (C) and 11.99 ± 0.09 mg O_2_ L^−1^ (MP). There were no significant differences between MP and C treatments (Fig. S3). In dark conditions (ES2), oxygen concentrations declined slightly over time in both control and MP samples, with final MP treatment concentrations being slightly lower (n = 24, *p* = 0.039, Fig. S3).

**Figure 2:**
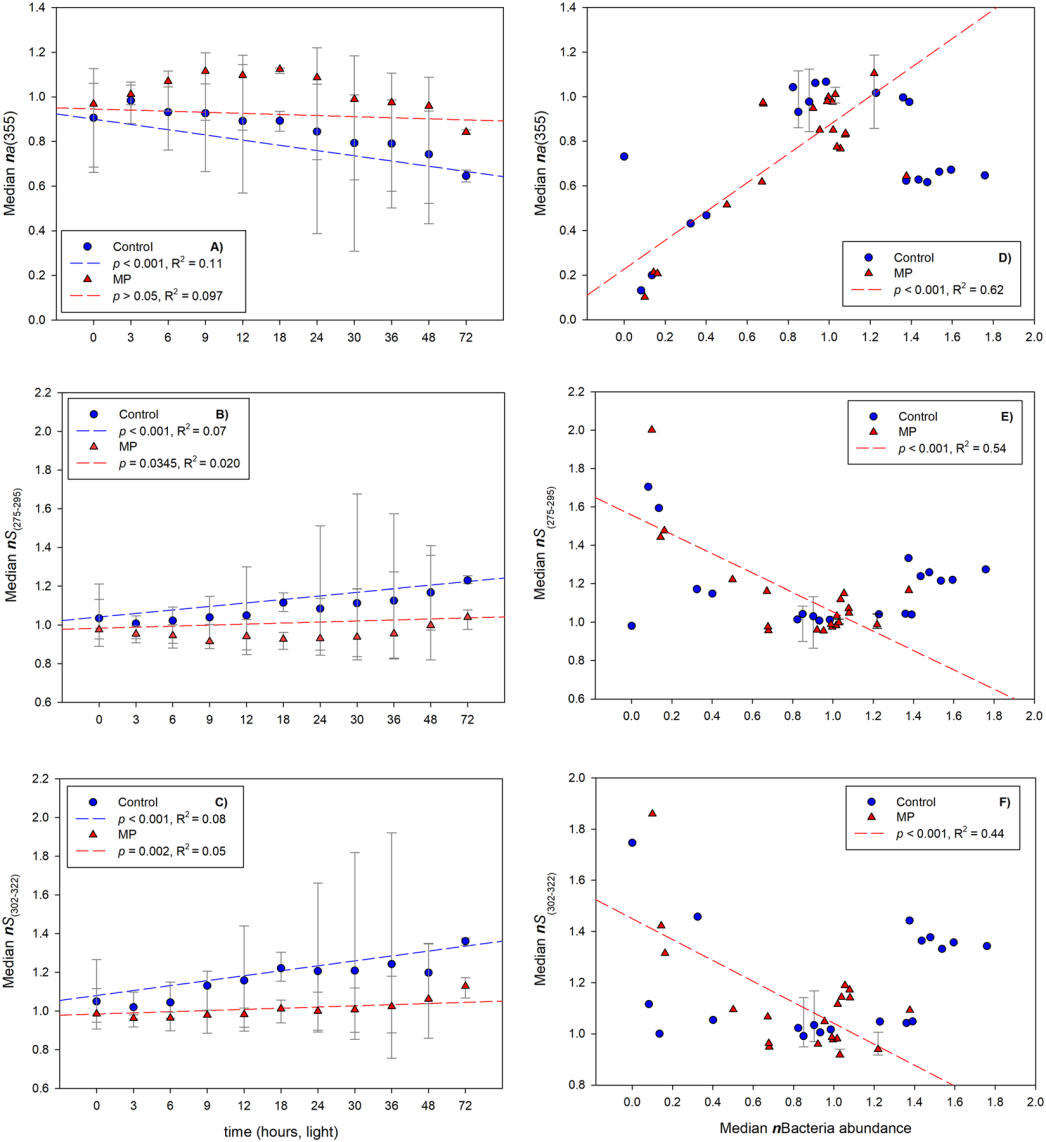
Regression of normalized CDOM absorbance (*a*355) and spectral slopes (*S*_275–295_, *S*_302–322_) with increasing light exposure time (left panel) and bacterial abundance (right panel) for control (C) and microplastics (MP) treatments in ES1. No dependency on bacterial abundance was observed in controls for *a*355 (r^2^ = 0.08, *p* > 0.05, D), and spectral slopes *S*_275–295_ (r^2^ = 0.00, *p* > 0.05, E), and *S*_302–322_ (r^2^ = 0.00, *p* > 0.05, F).

The CDOM absorption coefficient at 355 nm, *a*(355) m^−1^, declined linearly with increasing light exposure in both ES1 and ES2 (*p* < 0.0001). In ES1, with average *a*(355) values typical of shallow shelf waters, enclosed basins and river outflows of 1.81 ± 0.26 m^−1^ (C) and 2.14 ± 0.21 m^−1^ (MP) (ES 1a), 2.06 ± 1.04 m^−1^ (C) and 2.16 ± 0.80 m^−1^ (MP) (ES 1b), and 0.65 ± 0.08 m^−1^ (C) and 0.73 ± 0.06 m^−1^ (MP) (ES 1c), there was a higher CDOM loss rate in C with respect to MP samples (Fig. 2A). In ES2-light, *a*(355) averaged 6.57 ± 1.00 m^−1^ (C) and 6.67 ± 1.02 m^−1^ (MP), which is typical of inland eutrophic waters. Similarly to ES1, *a*(355) in ES2-light decreased with exposure both in the C and the MP samples (Fig. S1), with no significant difference between loss rates. In ES2-dark, *a*(355) increased in both treatments (Fig. S1), with no significant differences between C and MP (*p* > 0.05).

In both ES1 And ES2, we found a significant relationship between CDOM absorbance at 355 nm, *a*(355), and microbial abundance (ES1) or microbial activity (ES2). In ES1, we observed a significant increase in CDOM with increasing bacterial abundance in MP samples (Fig. 2D), with no clear trend in C. In ES2, an increase in CDOM *a*(355) was associated to a decrease in oxygen in the dark (r^2^ = 0.33, *p* = 0.002). In the light, CDOM *a*(355) was similarly inversely related to oxygen concentration (r^2^ = 0.83, *p* < 0.001), with no differences between C and MP. This relationship in light conditions can be explained by the fact that CDOM loss due to photodegradation was not sufficiently counterbalanced by microbial CDOM production whereas oxygen increased due to autotrophic production.

CDOM spectral slopes and CDOM absorption ratios were used to explore CDOM degradation, humification, aromaticity, and origin. We used the ratio of absorption 250 to 365 nm (*E*_2_:*E*_3_) as inversely related to average molecular weight, as high molecular weight CDOM strongly absorbs at longer wavelengths^25^. The ratio of absorption at 465 to 665 (*E*_4_:*E*_6_) was used instead to indicate CDOM humification and aromaticity^26,27^. Spectral slopes *S*_(275–295)_ and *S*_(302–322)_ were used to examine CDOM degradation and/or production of new organic material. Generally, lower *S* is related to the presence of higher molecular weight organic matter, comparatively indicating a CDOM with lower degree of degradation. Higher *S* indicates lower molecular weight organic material, with a typically higher degree of photochemical or microbial degradation.

**Figure 3:**
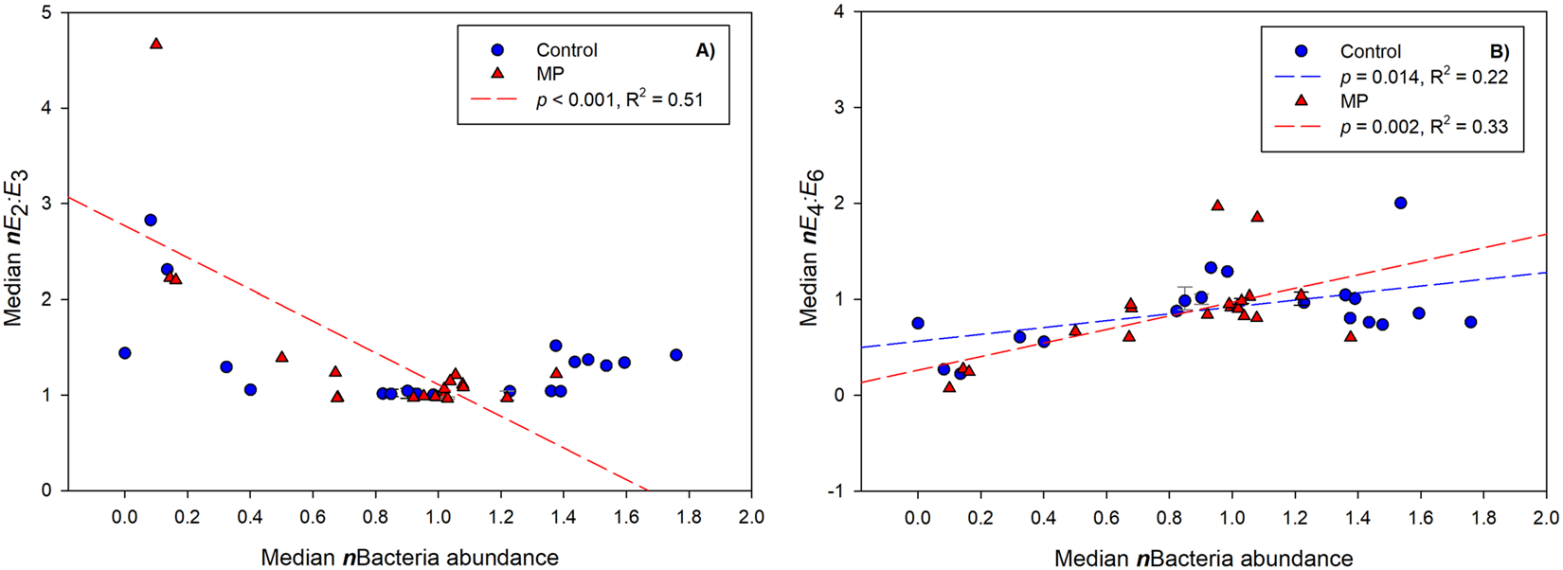
Regression analysis of normalized data of *E*_2_:*E*_3_ (absorption ratio at 250 nm and 365 nm) and *E*_4_:*E*_6_ (absorption ratio at 465 nm and 665 nm) with bacterial abundance, measured at t_0_ (start of the experiments) and t_f_ (end of the experiments, after 48 hours for experiments ES1b and ES1c and 72 hours for experiment ES1a) for control (C) and microplastics (MP) treatments in ES1. No dependency on bacterial abundance was observed in controls for *E*_2_:*E*_3_ (R^2^ = 0.12, *p* > 0.05, A).

In ES1, spectral slope increased in both C and MP samples with increased exposure time (Fig. 2B,C). The increase in *S* was higher in the C samples with respect to the MP samples, indicating an increased degradation. In ES1, absorbance ratios *E*_2_:*E*_3_ and *E*_4_:*E*_6_ ratios did not change with increased light exposure but did change with bacterial abundance (Fig. 3): a significant relationship was observed for *E*_2_:*E*_3_ with bacterial abundance only in MP samples, and for *E*_4_:*E*_6_ in both C and MP, with significant differences in the rate of change in this ratio between the two C and MP treatments.

Likewise, changes in *S* were correlated with changes in bacterial abundance only in MP samples (*p* < 0.0001, Fig. 2E,F). DOC, on the other hand, showed no relationship with bacterial abundance.

In ES2-light, *S*_(302–322)_ increased with exposure time in both C and MP treatments, while in the dark, *S*_(302–322)_ increased with incubation time in the C samples only (Fig. S4). No change was observed in *S*_(275–295)_ in the dark treatments, while it increased in the light with no discernable differences between C and MP. In ES2-light, *E*_2_:*E*_3_ increased over time and light exposure time (r^2^ = 0.91, *p* < 0.001), with no differences between C and MP. The absorbance ratio *E*_4_:*E*_6_ instead showed a rapid change only in MP samples towards the end of the experiment (Fig. S5) (r^2^ = 0.24, *p* = 0.005). In ES2-dark, *E*_2_:*E*_3_ decreased over time (*p* = 0.005), but with no differences between C and MP. *E*_4_:*E*_6_ instead remained constant, showing no dependency on the time of incubation (Fig. S5).

**Figure 4:**
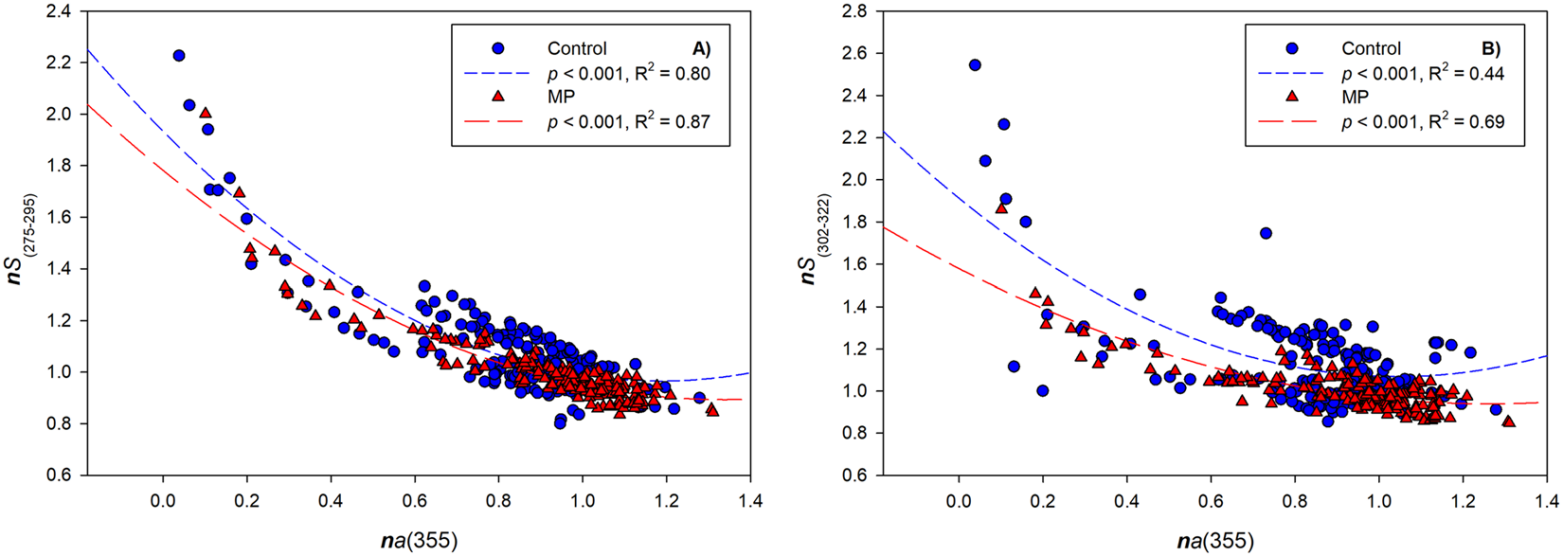
Exponential decay of normalized spectral slopes, *S*_275–295_ and *S*_302–322_ with changing CDOM absorbance *a*(355) for C (blue dots) and MP samples (red triangles) for control (C) and microplastics (MP) treatments in ES1.

Changes in CDOM absorption *a*(355) with respect to *S* indicate changes in the CDOM pool. As expected, *S* decayed exponentially with increasing absorbance in both C and MP treatments in ES1 (Fig. 4A,B). At higher CDOM concentration (and lower *S*), the organic matter pool might be characterized by compounds of higher molecular weight probably because of lower degradation or the generation of new organic matter. Significant differences (*p* < 0.002) were observed in the intercepts of the regression lines between C and MP samples, by fitting normalized *S*_(275–295)_ and *S*_(302–322)_ against the logarithm of normalized *a*(355). This suggests that, in the presence of microplastics, the baseline spectral slope *S* was lower for both *S* ranges as indicated by the following values:

**Table:** 

**C**	*S*_(275–295)_ = 0.99225–0.36560 *a*(355)	*S*_(302–322)_ = 1.0647–0.3030 *a*(355)
**MP**	*S*_(275–295)_ = 0.95522–0.36560 *a*(355)	*S*_(302–322)_ = 0.96597–0.3030 *a*(355)

In ES2-light, *S* and *a*(355) changed in a similar manner with no differences between C and MP (Fig. S6). In ES2-dark and only in MP samples, there was a significantly stronger (*p* < 0.001) decrease in *S*_(302–322)_ with increasing CDOM concentration, and a weaker dependency of *S*_(275–295)_ on *a*(355). No changes in both *S*_(302–322)_ and *S*_(275–295)_ with *a*(355) were observed in C (Fig. S6). In ES2-dark, again, the baseline spectral slope *S* was lower for both *S* ranges in the presence of microplastics as indicated by the following values:

**Table:** 

**C**	*S*_(275–295)_ = 1.1336–0.1113 *a*(355)	*S*_(302–322)_ = 1.0815–0.0747 *a*(355)
**MP**	*S*_(275–295)_ = 1.1314–0.1113 *a*(355)	*S*_(302–322)_ = 1.0789–0.0747 *a*(355)

## Discussion

Microplastics represent an emerging threat in marine and aquatic ecosystems in general. They are ubiquitous and could be considered as a new habitat for marine microorganisms^10,11^. Furthermore, microbial communities growing on microplastic particles modify physical properties of the particles themselves through biofilm creation, influencing their vertical transfer, residence time, and capacity to retain other contaminants^13^. As such, microplastics have a high potential to interfere with trophic processes and therefore with the export and cycling of organic matter^15^. In general, microplastics concentration is poorly defined because fragmentation processes of macro plastic debris leads to a continuum of sizes and forms. This has generated a mismatch between fragmentation rates and observed concentrations^5^ and a major challenge for modeling studies. At the moment, no “standard” microplastic reference particle exists.

The polystyrene microplastics used in our study are typical of the low-density polymers that are present in most marine surface waters^6,7,28^. The particles were inert over the time of our experiments with respect to CDOM release from the particles themselves. Although virgin standard plastic particles aren’t typical of marine environments, our results indicate that their presence as allochthonous particles can modify CDOM production. As biofilms commonly occur on most particles in the aquatic environment independently from the substrate, we expect that aged and weathered microplastic particles in real marine conditions, as well as natural inorganic particles (e.g. sand, clay) are likely to have similar effects on microbial CDOM release.

Marine phytoplankton can directly release CDOM, but the majority of *in-situ* CDOM production is generated by microbial degradation of DOM, which is qualitatively different in terms of chemistry and reactivity^29^. CDOM produced by marine bacteria is more recalcitrant and, although subject to photolysis, is more resistant to rapid microbial metabolism compared to more labile substrates^20^. In surface waters, near to the air-sea interface, CDOM may accumulate in the sea-surface microlayer^30^ alongside other dissolved and particulate organic components^31,32^, favoring the establishment of a more distinct bacterial community^31,33^ and being subject to photochemical transformations.

The dominating role of photo-degradation was clear in both experiments as CDOM absorbance and related DOC decreased with exposure time (Figs 1, 2 and S1). On the other hand, microbial activity has the potential for both CDOM production and degradation. Microbial CDOM release was most evident in ES1, with typical CDOM concentrations of many marine waters compared to the CDOM rich conditions explored in ES2. Modifications to CDOM spectra and spectral slopes were different in control and MP conditions: microplastic effects on *in-situ* CDOM production were seen in a reduced loss of DOC and a slower increase in spectral slope in light exposed MP samples (Figs 1 and 2) and in the dark MP samples (Fig. S4). This suggests that despite CDOM photodegradation (ES1) or microbial degradation (ES2, dark), microbial CDOM production counterbalanced CDOM loss in the presence of polystyrene microplastics. In this context, we cannot specify whether polystyrene particles facilitated the production of ex-novo CDOM or whether the changes in CDOM observed were the result of microbial reworking or organic material inducing changes in CDOM molecular weight.

Changes in spectral slope with increasing absorbance (ES1, Fig. 4), especially in dark conditions (ES2, Fig. S6) gave clearer insights into these dynamics. In both experiments, lower spectral slopes and higher CDOM absorbance in MP samples suggested the production of higher molecular weight CDOM that offset losses due to CDOM degradation. Lower dissolved oxygen concentrations in dark conditions in MP treatments (ES2) also suggested an active microbial community. The net increase in dissolved oxygen in the light conditions overwhelmed any difference in microbial related oxygen consumption.

A net CDOM accumulation in microbial incubations of phytoplankton-derived DOM may occurr^21^, as heterotrophic bacteria rapidly consume labile compounds and release more chemically complex DOM^34^. In our study, CDOM accumulation was observed only in the MP-amended treatments, suggesting that microplastic particles can stimulate microbial CDOM production (Fig. 2D), and that this newly-produced CDOM is not subsequently easily degraded (Fig. 2A). Similar bacterial abundances at the beginning and the end of the experiments (ES1) indicate a similar rate of cell growth and cell death. During rapid cell growth and cell death, bacterial DOM exudates can accumulate^35^. Microbial CDOM intermediates that accumulated during our experiments were qualitatively different from the beginning to the end.

As indices for molecular weight, changes in spectral slopes (*S*_(275–295)_ and *S*_(302–322)_) in the MP samples further corroborated these hypotheses. Furthermore, the rate of increase in the *E*_4_:*E*_6_ ratio, an inverse proxy for humification, relatively to increasing bacterial abundance was higher in MP treatments with respect to C. This again suggests a modification (increase) in the bacterial consumption of humic-like substances in the DOM exudates in the presence of MP (Fig. 4B). Humic substances can be both consumed and produced by marine bacteria^36,37^. In the more productive conditions of ES2, samples in dark conditions showed a decrease in dissolved oxygen concentration in both C (from 6.86 ± 0.08 mgL^-1^ to 6.45 ± 0.06 mgL^-1^) and MP samples (from 6.27 ± 0.08 mgL^-1^ to 6.13 ± 0.09 mgL^-1^). The loss of oxygen was higher in C, and a higher microbial activity was confirmed by a higher *S* with respect to MP samples (Fig. S4). In a recent study, an increase in CDOM (measured as fluorescent DOM, FDOM) was observed alongside microbial respiration (increased oxygen consumption)^38^. However, a reduced microbial respiration did not correspond to a proportional decrease in CDOM production, leading to a net CDOM accumulation. According to the authors^38^, microbial CDOM production over labile substrates is lower than the microbial turnover of semi-labile DOM. This suggests that *in-situ* CDOM (or FDOM) generation by bacterial degradation of semi-labile DOM is accompanied by lower metabolic oxygen consumption rates. This is a process that prevails in large parts of the oceans and that confirms the link between DOM lability and the rate of CDOM generation previously reported^21^.

In our study, CDOM accumulation in the dark MP-treatments (ES2) with a relatively reduced rate of oxygen consumption compared to C, suggests a microbial production of more recalcitrant CDOM over a less-labile/refractory substrate such as polystyrene micro particles. The results from both sets of experiments, ES1 and ES2, indicate that microbial turnover of phytoplankton generated DOM results in an accumulation of qualitatively different CDOM, confirming that heterotrophic metabolisms can be an important source of CDOM whose chemical complexity and availability depend on the substrate on which it is being formed.

## Conclusions

Low-density buoyant microplastic particles are often present at higher densities near or at the surface^39^, where an accumulation of polyethylene, polypropylene and polystyrene microplastics is well documented^6,7^. These polymer types are the most abundant in marine environments: chemical, biological and physical conditions, including increased exposure to solar UV radiation and oxygen favor their thermal and microbial degradation, with the resultant release of measurable amounts of climate-relevant gases such as methane and ethylene^40^ and modifications to particles’ shape, size and buoyancy^41^. Following UV exposure, polystyrene microplastics may undergo a reduction in molecular mass and density, making them more inclined to remain near the surface^42^.

In a simplified manner, our experiments simulated a system highly enriched in organic material and microplastics, and exposed to solar radiation in conditions as close as possible to reality. Our results suggest that despite the photodegradative loss of organic matter (CDOM), the presence of microplastics stimulate microbial CDOM release. This increased CDOM production results in a modified DOM composition with potential impacts on optical, physical and chemical properties of the marine surface layers. It should be noted that the increased CDOM production does not necessarily depend on the microplastics used in our experiments, but may result from an increase in the presence of particle surfaces, autochthonous or allochthonous. What differentiates microplastics is their relatively recent introduction and their growing presence in aquatic ecosystems, with global production of plastic expected to double in the next two decades^43^. As a result, marine environments, already affected by plastic pollution, will experience a progressive introduction of “new” substrates. The present study indicates that these new substrates have the potential to influence the biological production of carbon.

When extrapolated to the field, a microplastics-driven increase in surface CDOM could have multiple effects on marine systems and biological cycles. High CDOM concentrations modify the penetration of solar radiation, both for primary productivity as well as UV-related modifications of the biological community. Additionally, higher concentrations of surface organics imply changes in the air-sea CO_2_ flux and microbial respiration^44^. While a recent study explored plastics potential as carbon source for microorganisms^22^, our results indicate that microplastics, as inert particle substrates, also act as a support substrate for microbial carbon release. Thus, microplastics act as localized hotspots for high microbial activity, driving modifications to carbon cycling at the sea surface.

Further studies are needed to address microplastics-driven changes in the mechanisms of carbon capture, transformation and release in aquatic environments and how such changes differentiate microplastics effects from those of natural aquatic particles^45^. To facilitate this, it would be extremely helpful if a common standard for microplastic particles could be agreed upon, allowing for a more organized effort in their study and mitigation.

## Methods

### Microcosms experimental set up 1 (ES1)

Three replicate experiments were performed between August and November 2016. Two non-axenic strains of marine diatoms (*Chaetoceros socialis*, CCAP nr. 1010/19 and *Thalassiosira weissflogii*, CCAP nr. 1085/18) and a non-axenic strain of the coccolitophore *Emiliania huxleyi* (CCAP nr. 920/12) were obtained from the culture collection of the Scottish Association for Marine Sciences (SAMS) and grown in Guillard’s f/2 + Si medium (for diatoms) and f/20 (*E. huxleyi*)^46^ prepared from artificial seawater (ASW) according to the Marine Biological Laboratory of Woods Hole recipe nr. 1^47^. Molar concentrations of the added salts in 1 L Milli-Q water were: NaCl (423 mmol L^−1^), KCl (9 mmol L^−1^), CaCl_2_.2H_2_O (9.27 mmol L^−1^), MgCl_2_.6H_2_O (22.94 mmol L^−1^), MgSO_4_.7H_2_O (25.5 mmol L^−1^), NaHCO_3_ (2.14 mmol L^−1^). Prior to use, the ASW was filtered through a 0.2 µm cartridge (Whatman Polycap capsule filter) and autoclaved. The f/2 + Si and f/20 media were also filtered through 0.2 µm cartridge and autoclaved. The cultures were grown in 2 L. flasks on a 12:12 light/dark cycle (light intensity 54.4±0.12 µmol m^−2^ s^−1^) between 18 °C and 20 °C and reached abundances of 1.2 × 10^4^ cells mL^−1^ (*C. socialis*), 3.7 × 10^4^ cells mL^−1^ (*T. weissflogii*) and 1.7 × 10^4^ cells mL^−1^ (*E. huxleyi*). The phytoplankton cell abundance was measured microscopically and calibrated with optical density (OD) measurements at 420 nm (*C. socialis*) 684 nm (*T. weissflogii*) and 675 nm (*E. huxleyi*) by analyzing the growth curves. After the growth phase, each culture was filtered through a 1.0 µm membrane to remove phytoplankton cells while maintaining the original concentrations of dissolved organic matter (DOM) and bacteria. The filtrate (400 mL) of exudates and bacteria was equally distributed in four microcosms each containing 4 L of filtered and autoclaved ASW, with a density of 1.022 g/cm^3^ and a salinity of 30 PSU. An aqueous solution of 30 µm diameter transparent polystyrene microbeads (Sigma-Aldrich, nr. 84135) with a density of 1.05 g/cm^3^ t was added to two microcosms to a final concentration of approximately 2200 microplastic particles L^-1^. Two other microcosms served as controls without microplastic particles. All four microcosms were covered and aerated for 90 hours to insure mixing of the ASW, microplastic particles and filtrate. After incubation, water samples from each control (C) and microplastic containing (MP) microcosms were used to fill 12 quartz cuvettes (10-cm pathlength Hellma 120-QS, Quartz SUPRASIL).

8 cuvettes (4 C, 4 MP) were exposed to a light source with a pseudo solar spectrum (Philips OSRAM Metal halide lamp, HQI-TS, 150 W/D). Exposition was measured continuously at the surface of the 8 cuvettes in four wavelengths (with a 10-nm waveband and centre wavelengths of 380, 440, 590 and 670) (Skye Instruments). 4 cuvettes (2 C, 2 MP) were kept in the dark for the duration of each experiment. All cuvettes were maintained at the same temperature (26 ± 2 °C). CDOM absorbance was monitored from each cuvette in specific time frames (Table 1). Bacterial abundance was determined for each cuvette at the beginning (t_0_) and the end of the experiment (t_f_) and samples were fixed with 25% GDA and stored at −20 °C until analysis.

**Table 1:** Experimental conditions for first experiment (ES1) series.

Experiment	Specie	Cell abundance (10^4^ mL^−1^)	Duration (hours)	CDOM measurements (hours)
ES 1a	*T. weissflogii*	3.72	72	0, 3, 6, 9, 12, 18, 24, 30, 36, 48, 72
ES 1b	*C. socialis*	1.16	48	0, 3, 6, 9, 12, 24, 30, 36, 48
ES 1c	*E. huxleyi*	1.66	48	0, 3, 6, 9, 12, 24, 30, 36, 48

Each experiment was repeated with a different monoculture.

### Microcosms dark/light experimental set up 2 (ES2)

To explore the possible influence of phytoplankton exudates on CDOM and bacteria, a solution of concentrated exudates from a mixed culture (*C. socialis* and *T. weissflogii*, in equal concentrations) was used in dark or pseudo-sunlight conditions. The mixed exudate was filtered through GF/F 0.7 μm glass fiber filters to remove phytoplankton and larger particles. The filtrate was distributed in 12 10-cm quartz cuvettes (Hellma 120-QS, Quartz SUPRASIL), adding a concentrated solution of polystyrene microbeads to 6 of the cuvettes (2600 microspheres per cuvette, corresponding to approximately 92000 microplastic particles L^-1^), with 6 cuvettes used as a control (no microplastic addition). Oxygen concentration changes were used as a proxy of microbial activity (consumption) and oxygen was measured at the beginning and at the end of each 96-hour test (FireSting Oxygen optical probe and temperature sensor, PyroScience®). CDOM concentration was measured at t_0_ (beginning), and every 24 hours (t_24_, t_48_, t_72,_ t_96_) (Table 2). The experiment was run twice, once in conditions of continuous illumination and another in conditions of total darkness. In the first test, 12 cuvettes (6 C, 6 MP) were exposed to the same light source as ES1, to a constant temperature (26 ± 2 °C) for 96 hours. In the dark test, 12 cuvettes (6 C, 6 MP) were kept in the dark at constant temperature (26 ± 2 °C) for 96 hours (Table 2).

**Table 2:** Experimental conditions for the second experiment (ES2) series.

Experiment	Exudates	Duration (hours)	MP abundance(mL^−1^)	CDOM measurements (hours)	Oxygen measurements (hours)
ES 2 light	*T. weissflogii C. Socialis*	96	92	0, 24, 48, 72, 96	0, 96
ES 2 dark	*T. weissflogii C. Socialis*	96	92	0, 24, 48, 72, 96	0, 96

### Chromophoric Dissolved Organic Matter (CDOM) optical measurements

CDOM absorbance was measured using a Lambda 10 UV-Visible Spectrophotometer (Perkin Elmer) from 210 to 750 nm at 960 nm/min, at 1 nm wavelength resolution, at room temperature (20 °C) and corrected for Milli-Q water each day of analysis. Spectra were corrected for scattering by subtracting the absorbance values at 700 nm. Absorption coefficients *a*(λ) were calculated from absorbance (A_λ_) values as^48^:

1 $$a(\lambda ),{{\rm{m}}}^{-1}=2.303{{\rm{A}}}_{\lambda }/{\rm{L}}$$

where L is the path length of the cuvette (0.10 m).

As CDOM absorption is exponential, the absorption spectral slope *S* (nm^−1^) was determined by a standard equation by linear regression of log-transformed absorption spectra against the wavelength^48^:

2 $$a(\lambda )={a}_{0}{e}^{-S(\lambda -{\lambda }_{0})}$$

With *a*(λ_0_) being the absorption coefficient at a reference wavelength λ_0_. We used multiple 20-nm wavelength intervals in a stepwise (1 nm) linear regression analysis according to Loiselle *et al*.^49^. *S* computed between 275 and 295 nm, *S*_(275–295)_, and *S* in the interval 302–322 nm, *S*_(302–322)_ showed the highest correlation to CDOM absorption at 355 nm (*r*^2^ > 0.97) while also allowing for comparison to other studies^26^. The wavelength range 275–295 nm has been shown to correlate with changes in CDOM due to irradiation (photobleaching) as well as being inversely related with DOM molecular weight^26^. Relevant spectra for ES1 and ES2 are included in the supplementary information.

### Blank control

To exclude any possible release of CDOM from polystyrene particles under pseudo-sunlight irradiation, we performed a test filling six 10-cm quartz cuvettes (Hellma 120-QS, Quartz SUPRASIL) with Milli-Q water, and adding a concentration of 92000 particles per Liter to three of the cuvettes (about 2600 particles per cuvette), leaving three as control. CDOM concentration was measured from 200 to 750 nm at 960 nm/min, at 1 nm wavelength resolution, at room temperature (20 °C) at t_0_ (beginning), and every 24 hours (t_24_, t_48_, t_72,_ t_96_). No significant differences between controls and treatments, as well as between beginning and end, were detected for the wavelengths used in the ES1 and ES2 experiments (250–700 nm, paired t-test per every wavelength, Fig. S7). Significant differences (*p* < 0.05) between control and treatments were observed after 96 hours only below 250 nm. Given the short time of these experiments, it seems unlikely that there was sufficient exposure to create structural changes in the particles which might lead to leaching.

### Dissolved Organic Carbon (DOC) concentrations

DOC dynamics were estimated by comparing measured DOC concentrations with CDOM absorption following Fichot and Benner^50^. Replicate samples (5) from each monoculture were exposed to the same experimental conditions as ES1 and ES2 for 4 hours. Samples were removed hourly (t_0_ - t_240_) for CDOM absorption, and then acidified with 80 μL of 85% phosphoric acid and stored in the dark at 4 °C for DOC analysis. DOC measurements were performed in 4 replicate on each sample using high-temperature catalytic oxidation (TOC-VCSH, Shimadzu) following Sugimura and Suzuki^51^. The calibration with CDOM spectra was performed by following a linear relationship between spectral slope *S* and the ratio of *a*_g_(λ)/DOC^50^:

3 $$\mathrm{ln}[DOC]=\alpha +\beta \,\mathrm{ln}\,[{a}_{g}({{\rm{\lambda }}}_{1})]+\gamma \,\mathrm{ln}\,[{a}_{g}({{\rm{\lambda }}}_{2})]$$

Where *α*, *β*, and *γ* are regression coefficients for 329 nm *a*_g_(λ_1_) and 349 nm *a*_g_(λ_2_), following the best model fit (Table 3).

**Table 3:** Results of the linear regression analysis between DOC and CDOM spectral slope samples.

	r^2^	a_g_(λ_1_), nm	a_g_(λ_2_), nm	α	β	γ
Exudates	0.74	329	349	2.7800	8.6733	−6.9378

### Bacterial abundance

Samples for bacterial abundance were collected at the beginning and at the end of each experiment. Samples (1.5 mL) from each cuvette were fixed with 60 μL glutaraldehyde (25% final concentration) and stored at −20 °C until flow cytometry analysis. Samples were stained with SYBR Green I (Molecular Probes) and yellow-green latex beads (Polyscience, 0.5 μm) were used as an internal standard. Bacteria were enumerated by detecting their signature at 488 nm excitation in a plot of side scatter (SSC) versus green fluorescence (FL1) in a Becton & Dickinson FACSCalibur flow cytometer^52^.

### Data analysis and statistics

Data for each parameter, in all experiments (ES1 and ES2), all measurements (CDOM, DOC, dissolved oxygen, bacteria cell number) were normalized to the conditions of all samples at the start of the experiment (t_0_), to allow for comparison of results across replicate experiments, according to the following equation:

4 $${\rm{n}}{d}_{{\rm{i}}}{{\rm{t}}}_{{\rm{j}}}={d}_{{\rm{i}}}{{\rm{t}}}_{{\rm{j}}}/({\rm{average}}\,{d}_{1\to 12}{{\rm{t}}}_{0})$$

where n*d* is the normalized parameter for the *i*^*th*^ at time t_j_ where *j* = hours (0, 3, etc. to 48 or 72).

Statistical analysis on normalized data (linear regression and Mann-Whitney Rank Sum tests) was performed with Prism7.03 (GraphPad) and Minitab® 18.1, accepting a statistical significance of *p* < 0.05.

## Electronic supplementary material

Supplementary Information

## Data Availability

All data are available upon request and will be uploaded on the Pangaea database https://www.pangaea.de after publication of the manuscript, in conformity with the requirements of all Horizon2020 funded research projects.
