# Supplementary material for: Polystyrene microplastics increase microbial release of marine Chromophoric Dissolved Organic Matter in microcosm experiments
Source: Sci Rep. 2018 Oct 2;8:14635. doi: 10.1038/s41598-018-32805-4 (PMC6168505; doi:10.1038/s41598-018-32805-4)
Supplement: Supplementary file 1 — Supplementary Information [file 41598_2018_32805_MOESM1_ESM.pdf]

Supplementary material to the manuscript:

"Polystyrene microplastics increase microbial release of marine Chromophoric Dissolved Organic Matter in microcosm experiments"

Authors: Luisa Galgani, Anja Engel, Claudio Rossi, Alessandro Donati and Steven A. Loiselle

Tables

Table S1: Estimated DOC concentrations in ES1 and ES2

**ES1: experiments**	**Average DOC (µMol L^{-1})**	**Linear Regression Coefficient (µMol L^{-1} h^{-1})**
ES 1a (Thalassiosira weissflogii, C)	250.3 ± 34.8	- 1.24 ± 0.15
ES 1a (Thalassiosira weissflogii, MP)	246.4 ± 29.5	- 0.17 ± 0.18
ES 1b (Chaetoceros socialis, C)	452.8 ± 360.3	- 13.3 ± 2.7
ES 1b (Chaetoceros socialis, MP)	357.3 ± 166.4	- 3.31 ± 1.40
ES 1c (Emiliania huxleyi, C)	76.3 ± 15.0	- 0.50 ± 0.11
ES 1c (Emiliania huxleyi, MP)	81.5 ± 10.7	- 0.48 ± 0.07

**ES2: experiments dark/light**	**Average DOC (10^3 * µMol L^{-1})**	**Linear Regression Coefficient (µMol L^{-1} h^{-1})**
Dark, C	31.8±0.6	+ 10.9 ± 2.4
Dark, MP	31.5±1.0	+ 11.5 ± 5.0
Light, C	21.6±5.0	- 126.1 ± 15.1
Light, MP	22.0±5.2	- 130.2 ± 15.1

**In ES2, estimated DOC concentrations were extremely high, indicating the high load of organic material and exudates from the cultures.*

Figures

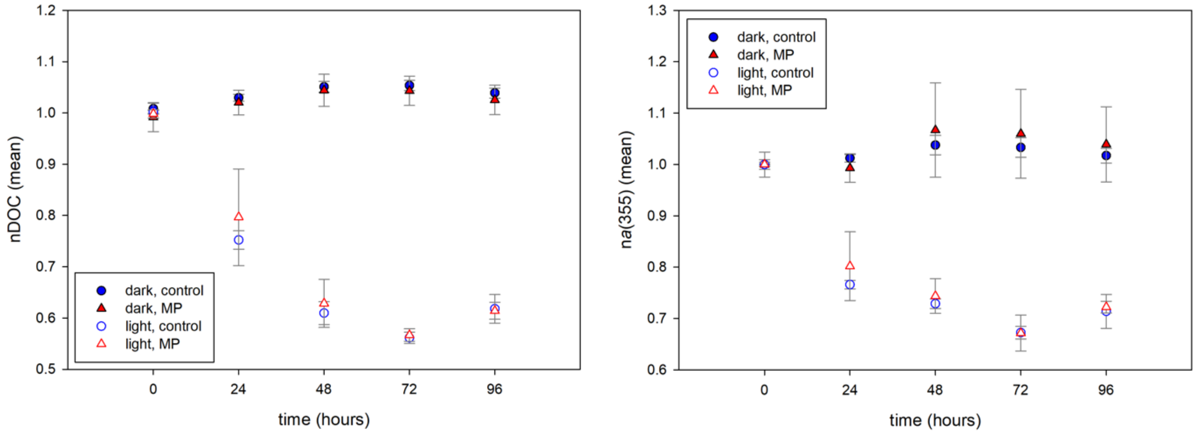

Figure S1. (left) Changes in estimated and normalized DOC concentrations (median) over increased exposure (light) and time (dark). (right) Changes in median normalized CDOM absorption measured at 355 nm (a(355), m^{-1}) over time for control (C) and microplastics (MP) treatments in ES2 (n = 30).

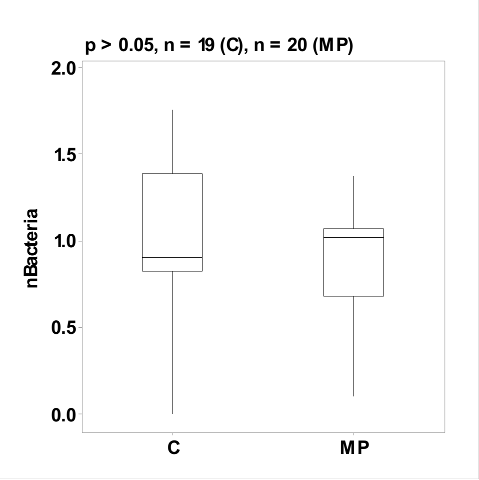

Figure S2. Comparison of bacterial abundance for control (C) and microplastics (MP) treatments in ES1. Data have been normalized to compare ES1a, ES1b and ES1c.

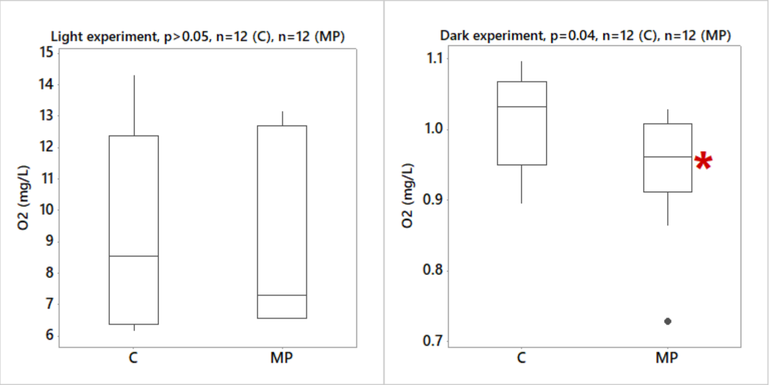

Figure S3. Comparison of dissolved oxygen concentrations in light and dark experiments, measured at the beginning and at the end of each test (t_0, t_{96}) for control (C) and microplastics (MP) treatments in ES2 (n = 24).

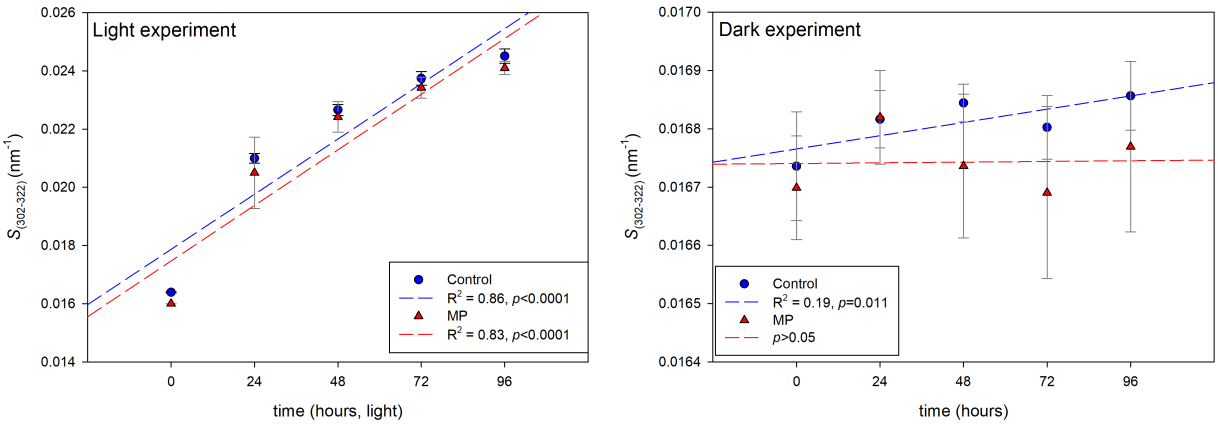

Figure S4. Changes in median spectral slopes values, $S_{(302\text{-}322)}$, with increased exposure (light) and time (dark) for control (C) and microplastics (MP) treatments in ES2 (n = 30). In the dark, no dependency on time was observed $S_{(302\text{-}322)}$ in MP samples ($R^2 = 0.00$, $p > 0.05$).

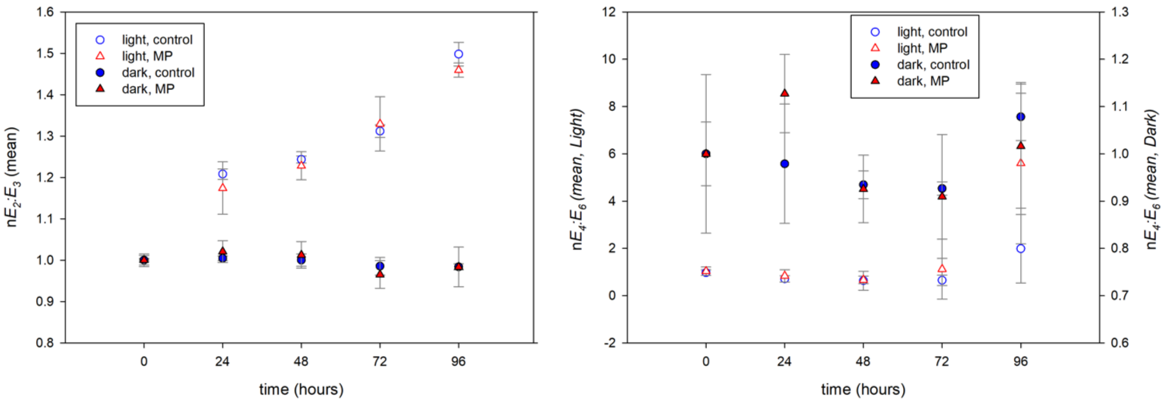

Figure S5. ES2. Normalized mean absorbance ratios, E_2:E_3 (ratio of absorbance measured at 250 to 365 nm) and E_4:E_6 (ratio of absorbance measured at 465 to 665 nm) over time for control (blue) and microplastics (MP, red) treatments in both light and dark experiments (n = 30).

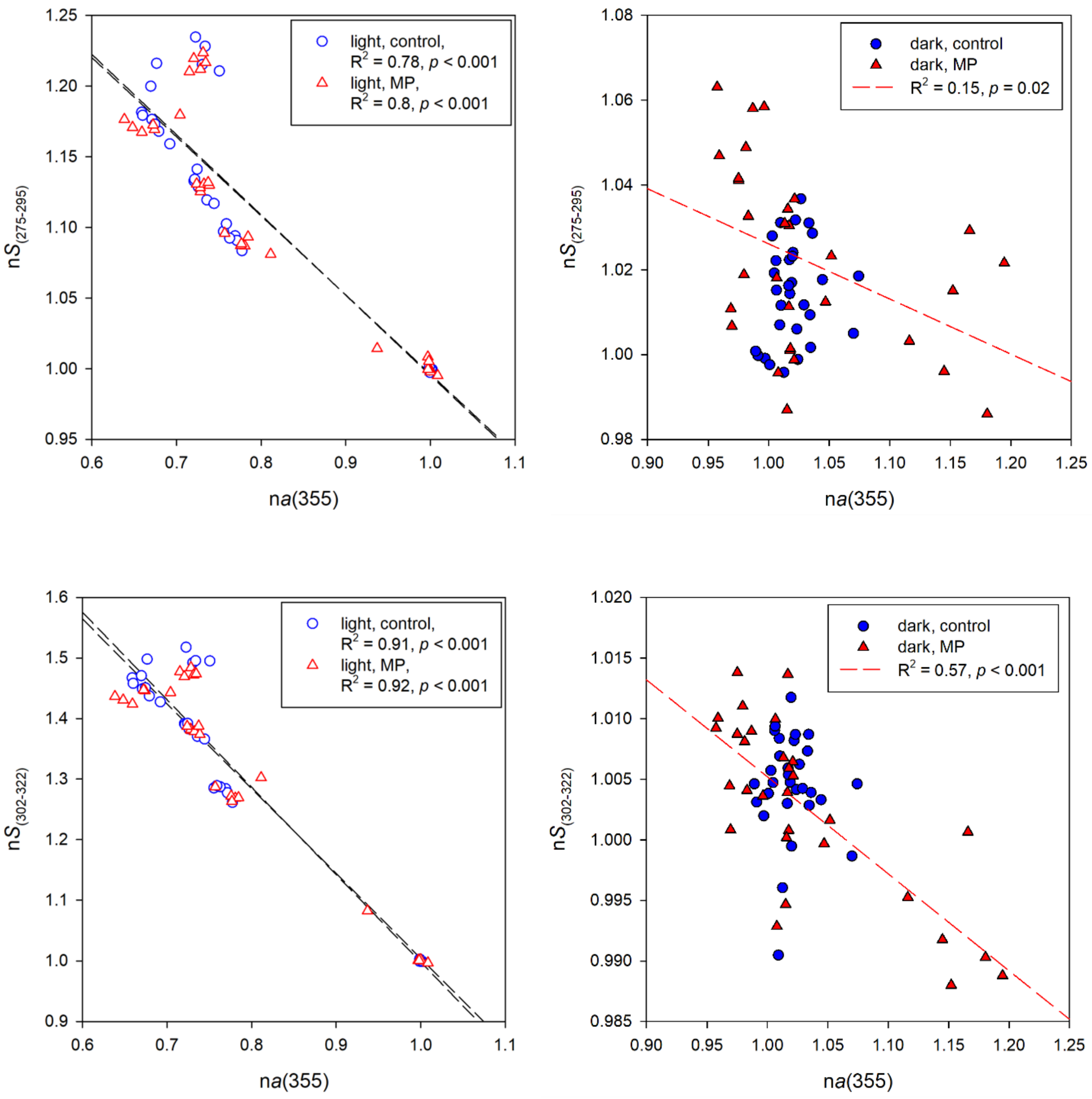

Figure S6. Changes in spectral slopes $S_{(275\text{-}295)}$ and $S_{(302\text{-}322)}$ with increasing CDOM concentration (absorbance measured at 355 nm, a(355)), in light and dark experiments of ES2. Data have been normalized. In the dark-controls (C), no dependency on a(355) nm was observed for any of the spectral slopes $S_{(275\text{-}295)}$ (R^2 = 0.00, p > 0.05) or $S_{(302\text{-}322)}$) (R^2 = 0.00, p > 0.05).

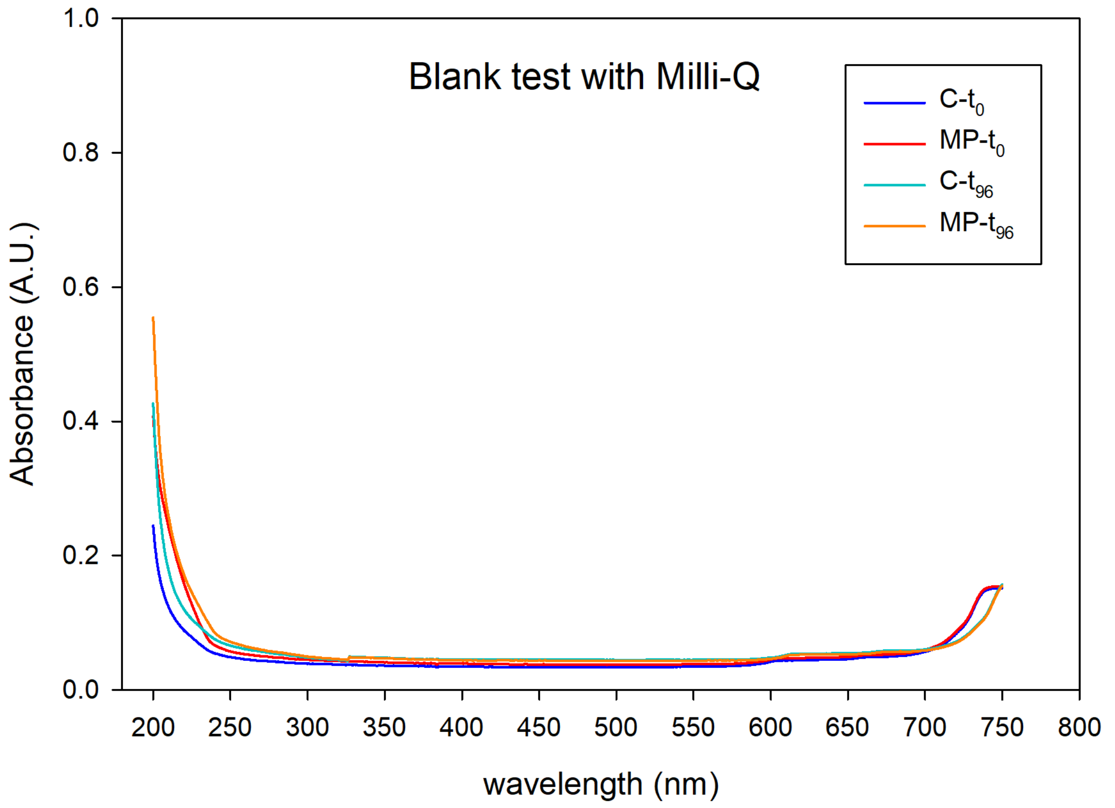

Figure S7. Spectra of the blank test at time 0 and after 96 hours. C = control, MP = microplastics treatments. All cuvettes were filled with Milli-Q water.

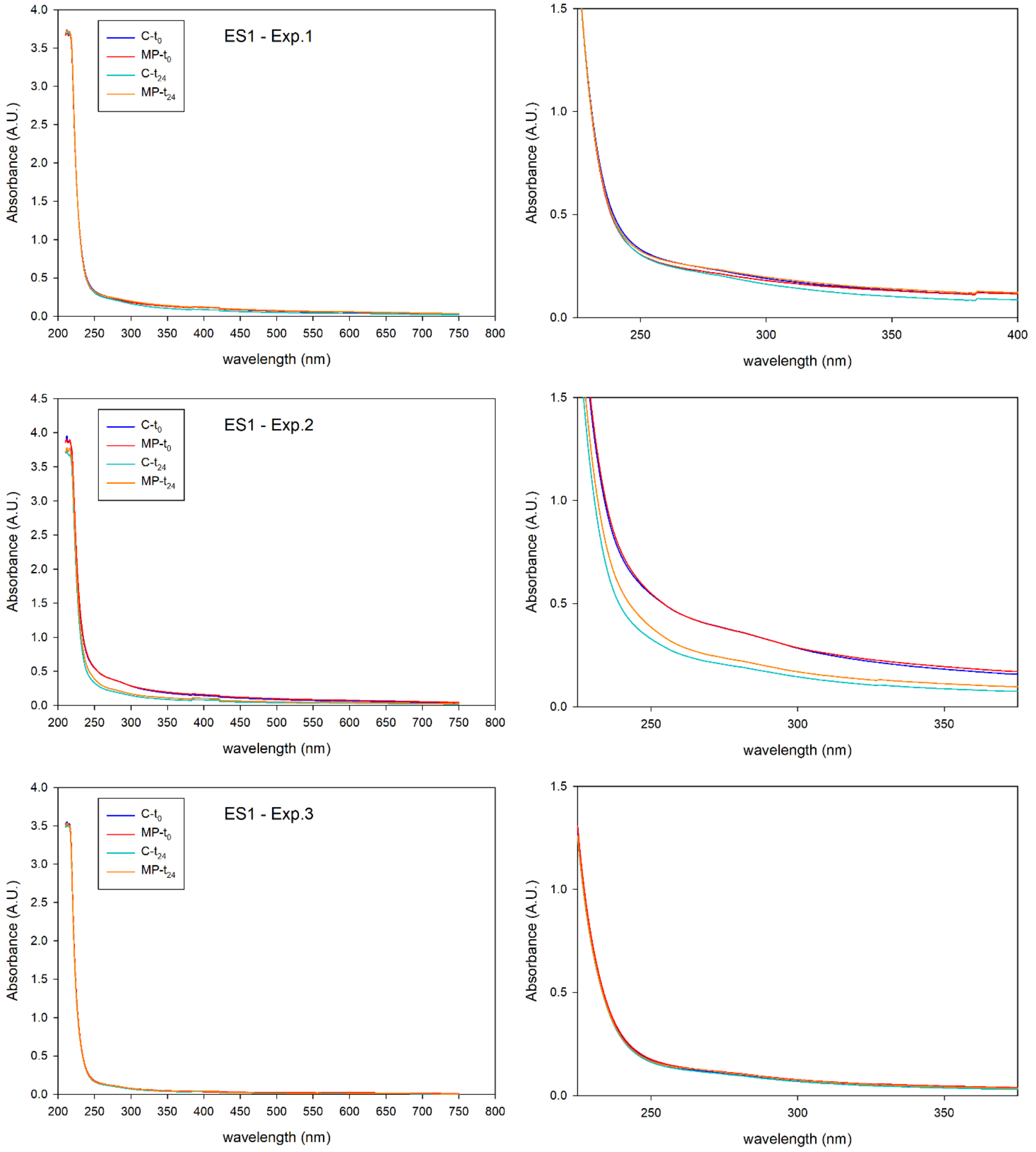

Figure S8. Spectra at time 0 and after 24 hours for ES1 – experiment 1, 2 and 3. On the left side, full spectra. On the right side, close-up between 225 and 375 nm. All cuvettes contained phytoplankton exudates and bacteria (C, control) and ~2200 polystyrene microparticles per liter (MP, treatments).

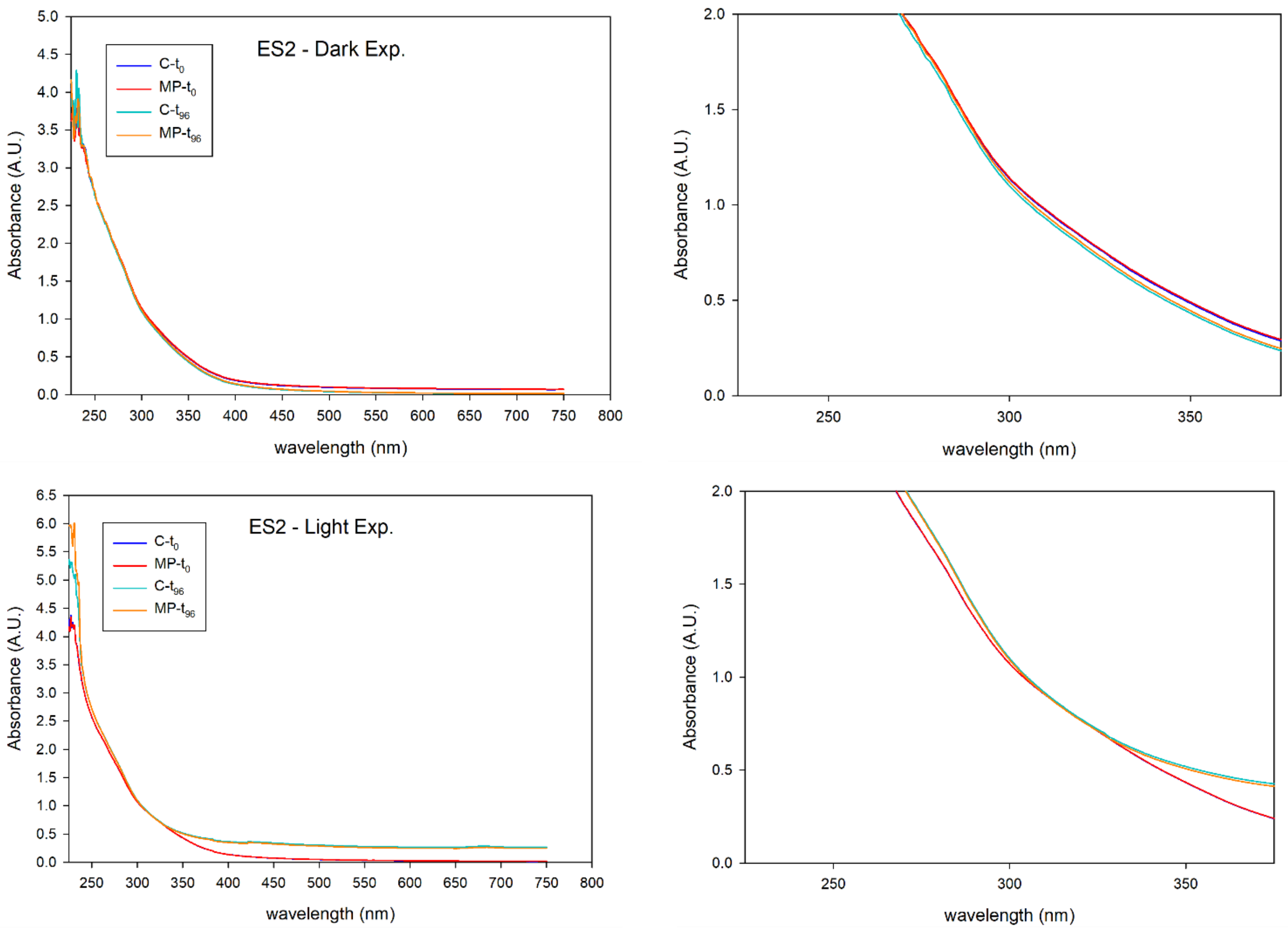

Figure S9. Spectra at time 0 and after 96 hours for ES2 – experiment dark and light. On the left side, full spectra. On the right side, close-up between 225 and 375 nm. All cuvettes contained phytoplankton exudates and bacteria (C, control) and ~2600 polystyrene microplastics per treated cuvette (MP, treatments), corresponding to ~9200 polystyrene microparticles per liter.
